# Scale-dependent bi-trophic interactions in a semi-arid savanna: how herbivores eliminate benefits of nutrient patchiness to plants

**DOI:** 10.1007/s00442-016-3627-0

**Published:** 2016-04-19

**Authors:** Cornelis van der Waal, Hans de Kroon, Frank van Langevelde, Willem F. de Boer, Ignas M. A. Heitkönig, Rob Slotow, Yolanda Pretorius, Herbert H. T. Prins

**Affiliations:** Resource Ecology Group, Wageningen University, Droevendaalsesteeg 3A, 6708 PD Wageningen, The Netherlands; Department of Experimental Plant Ecology, Institute for Water and Wetland Research, Radboud University, PO Box 9010, 6500 GL Nijmegen, The Netherlands; School of Life Sciences, University of KwaZulu-Natal, Westville Campus, Private Bag X54001, Durban, 4000 South Africa; Centre for Wildlife Management, University of Pretoria, Private bag X20, Hatfield, Pretoria, South Africa; Agri-Ecological Services, PO Box 28, Omaruru, Namibia

**Keywords:** Tree–grass, Nutrients, Trophic cascade, Grazing, Allometric scaling theory

## Abstract

The scale of resource heterogeneity may influence how resources are locally partitioned between co-existing large and small organisms such as trees and grasses in savannas. Scale-related plant responses may, in turn, influence herbivore use of the vegetation. To examine these scale-dependent bi-trophic interactions, we varied fertilizer [(nitrogen (N)/phosphorus (P)/potassium (K)] applications to patches to create different scales of nutrient patchiness (patch size 2 × 2 m, 10 × 10 m, or whole-plot 50 × 50 m) in a large field experiment in intact African savanna. Within-patch fertilizer concentration and the total fertilizer load per plot were independently varied. We found that fertilization increased the leaf N and P concentrations of trees and grasses, resulting in elevated utilization by browsers and grazers. Herbivory off-take was particularly considerable at higher nutrient concentrations. Scale-dependent effects were weak. The net effect of fertilization and herbivory was that plants in fertilized areas tended to grow less and develop smaller rather than larger standing biomass compared to plants growing in areas that remained unfertilized. When all of these effects were considered together at the community (plot) level, herbivory completely eliminated the positive effects of fertilization on the plant community. While this was true for all scales of fertilization, grasses tended to profit more from coarse-grained fertilization and trees from fine-grained fertilization. We conclude that in herbivore-dominated communities, such as the African savanna, nutrient patchiness results in the herbivore community profiting rather more than the plant community, irrespective of the scale of patchiness. At the community level, the allometric scaling theory’s prediction of plant—and probably also animal—production does not hold or may even be reversed as a result of complex bi-trophic interactions.

**Electronic supplementary material:**

The online version of this article (doi:10.1007/s00442-016-3627-0) contains supplementary material, which is available to authorized users.

## Introduction

In natural systems, resources such as water and nutrients are heterogeneously distributed in space and time on a resource-specific scale (Cromsigt et al. 2009; Jacobs et al. 2007). The patchy distribution of these resources has important consequences for the productivity and distribution of plants. The local scale of patchiness (grain size) at which the same amounts of nutrients are supplied to plants has been shown to affect the resource assimilation and performance of single plants (e.g. Felderer et al. 2013; Kume et al. 2006), plant competitive ability (Fransen et al. 2001; Mommer et al. 2012), species composition and structure of experimental plant communities (Hutchings et al. 2003; Wijesinghe et al. 2005; Xi et al. 2015) and leaf nutrient content of trees (van der Waal et al. 2011a). Soil heterogeneity is likely to affect the responses of plant communities to environmental change (Garcia-Palacios et al. 2012).

Changes in these plant properties (productivity, structure, food quality for herbivores), in turn, are expected to affect higher trophic levels (i.e. consumers). Experimental manipulation of the spatial arrangement of forage resources has demonstrated that herbivory may be strongly influenced by the scale of forage patchiness (Cromsigt and Olff 2006, 2008; Pretorius et al. 2011). However, to our knowledge, whether soil heterogeneity has effects on herbivore utilization has not been studied. Herbivory feedback affects plant competition and co-existence, although most evidence of this effect originates from studies with invasive plant species (Heard and Sax 2013; Orrock et al. 2010, 2015; Ruifrok et al. 2015). It is therefore questionable whether the reported effects of soil heterogeneity on plant populations and communities hold in the presence of their natural herbivores. Here we report on a field fertilization experiment in an African savanna which was designed to test how the scale of patchily distributed soil resources affects tree–grass growth and browser–grazer consumption. The design was unique in that it allowed the effects of patch quality (levels of fertilization) and scale (patch sizes) to be examined independently.

In savannas, local nutrient patches may scale from sub-meters in individual urine or dung patches, to meters in rhino dung middens, termitaria or beneath large-tree canopies, up to tens of meters in natural nutrient hotspots or abandoned livestock bomas (Belsky 1994; Grant and Scholes 2006; Schlesinger et al. 1996; van der Waal et al. 2011b). Scaling theory predicts that the grain size at which organisms perceive and respond to resource heterogeneity in their environment is determined by the size of the organism (Laca et al. 2010; Ritchie and Olff 1999). As such, large organisms are expected to consume coarser grain sizes of patchily distributed resources and small organisms to consume finer grain sizes (Ritchie and Olff 1999). This allometric scale dependence may, in turn, influence resource partitioning between co-existing organisms that perceive the spatial heterogeneity differently (Prins and Van Langevelde 2008). Following scaling theory, the smaller sized savanna grasses are expected to profit from soil resources distributed at relatively fine scales and the larger sized trees to profit from patches at relatively coarser scales. In support of this theory, savanna trees supplied with the same amount of nutrients in a single large patch showed increased leaf nitrogen (N) concentration relative to trees which received the same amount of nutrients but scattered over many small patches (van der Waal et al. 2011a). Viewed at the community level, when the same total amount of nutrients is supplied to an area—but at a different scale—we expect grasses to express superior nutrient uptake in relatively fine-grained environments and trees to express superior nutrient uptake in coarser grained environments.

Scale-induced changes in resource partitioning between trees and grasses may influence the foodscapes for large herbivores, which are expected to forage for high-quality plant material in a scale-dependent manner (Cromsigt et al. 2009; Laca et al. 2010). Large-sized herbivores, such as African elephant *Loxodonta africana*, for example, may not be able to respond to very fine (relative to body size) grain sizes (de Knegt et al. 2011; Laca et al. 2010). In particular, we expect that grazers may respond to nutrient enrichment at smaller patch sizes than browsers, as grass is expected to react to smaller scaled patches than trees. Such shifts in the grazer–browser balance may result in herbivore feedbacks that can affect the tree–grass balance (e.g. Roques et al. 2001; Sankaran et al. 2008), potentially modifying the initial effects of patchy fertilization. Feedbacks of herbivores on the vegetation have been shown to be an important determinant of vegetation structure in savanna systems (e.g. Asner et al. 2009; Kimuyu et al. 2014; van Langevelde et al. 2003).

In our experiment, nutrients were supplied in the form of nitrogen/phosphorus potassium (NPK) fertilizer at three different patch sizes to 50 × 50-m plots in an open savanna. The concentration of nutrients in the fertilized patches was varied in such a way that whole plots received fertilizer at three different loads with patches of different nutrient concentration and size. The three patch sizes and three levels of fertilization were chosen to mimic the range in patchiness from very local dung and urine patches (Orwin et al. 2009; Williams and Haynes 1995), to termite mounds (Joseph et al. 2013; Seymour et al. 2014) and kraals (van der Waal et al. 2011b). As argued by van der Waal et al. (2011a), the highest fertilizer concentration was realistic compared to nutrient concentrations measured in dung and urine patches.

Specifically we tested the following hypotheses (1) comparing within-patch nutrient concentrations, grasses are more responsive to nutrient supplies at smaller patches, and trees are responsive to nutrient supplies at larger patches; (2) in response to increasing within-patch nutrient concentrations, grazing intensity increases more at smaller patch sizes, while browsing intensity is expected to respond only at larger patch sizes; (3) with a constant nutrient load per plot, the herbaceous layer benefits relatively more when nutrients are supplied at the finer grain size and trees at the coarser grain size. We measured leaf N and P concentrations for the dominant tree species and two dominant grass species in this area, as leaf nutrient concentrations are the first to respond to fertilizer application. Leaf nutrient concentrations also underlie the increase in production and are a measure of forage quality to which consumers are known to respond (Pretorius et al. 2011; Prins and Beekman 1989; van Langevelde et al. 2008). We also measured biomass and tree growth, while grazer offtake was determined in a supplementary exclosure experiment.

## Methods

### Study area

The experiment was conducted in the Timbavati Private Nature Reserve (TPNR) in north-eastern South Africa. The TPNR borders the Kruger National Park’s western boundary, and fences between the game sanctuaries were removed in 1993, which enabled wildlife to move freely between the sanctuaries (Bigalke 2000).

January is the hottest month at the Satara weather station (50 km E of the study site), with a mean maximum temperature of 33.7 °C, and June is the coolest month, with a mean minimum temperature of 9.4 °C (Venter et al. 2003). The long-term mean rainfall (1983–2004) is approximately 450 mm (Ingwalala rainfall station, 5 km N of study site), with 78 % of rain falling between October and the end of March. The total annual (July to end June) rainfall varied over the study period: 351 mm during the 2004/2005 season, 433 mm during the 2005/2006 season, 393 mm during the 2006/2007 season and 273 mm during the 2007/2008 season.

The shallow soil of the study area is derived from granite, coarsely textured and nutrient poor (Venter et al. 2003). The woodland in the north-eastern part of the TPNR, where the experiment was conducted (24°14′12″S; 31°22′32″E) comprises a well-developed woody stratum dominated by short *Colophospermum mopane* trees (height <8 m) and a continuous herbaceous layer of medium height (<1 m) featuring species such as *Urochloa mosambicensis*,* Bothriochloa radicans*, *Digitaria eriantha*, *Brachiaria deflexa*, *Panicum maximum* (in order of dominance) and a variety of non-graminoid herbaceous species.

Large herbivore species commonly found in the study area include the African elephant *Loxodonta africana* (proportion of total spoor counts in the area, as an index of abundance: 10 %; Pretorius 2009), African buffalo *Syncerus caffer* (3 %), common duiker *Sylvicapra grimmia* (13 %), steenbok *Raphicerus campestris* (23 %), impala *Aepyceros melampus* (26 %), Burchell’s zebra *Equus burchellii* (7 %) and warthog *Phacochoerus aethiopicus* (6 %). Grazers such as the blue wildebeest *Connochaetes taurinus* and white rhinoceros *Cerathorium simum* and browsers such as the Greater kudu *Tragelaphus strepsiceros* and giraffe *Giraffa camelopardalis* were seen regularly but were present at low densities.

Fire in the TPNR is controlled, and the last fire at the study site occurred before 2004.

### Experimental setup

The experiment was laid out in an area that measured approximately 1 × 1.5 km. We followed a randomized block design consisting of thirty 50 × 50-m plots. Blocks, consisting of ten plots each, represented different topographical positions in the gently undulating landscape. Treatments followed an incomplete factorial design consisting of three factors: the scale of patchiness in which fertilizer was delivered to plots (three levels; Table 1), the within-patch fertilizer concentration (four levels) and the total fertilizer load each plot received (four levels). The three scale treatments consisted of different spatial configurations of fertilized patches: namely, the fertilizer distributed over the whole 50 × 50-m plot area, or concentrated in either five 10 × 10-m patches or twenty-five 2 × 2-m patches. Patches were randomly allocated within the block area. Each plot was surrounded by a buffer area of 25 m so that plots were at least 50 m apart. The within-patch fertilizer concentration levels were: 0 (control), 1.2, 6.0 and 30 g N m^−2^, respectively. Treatment levels were chosen to yield similar total fertilizer loads per plot along the diagonal formed by a scale of patchiness–within-patch fertilizer concentration table. This resulted in the following fertilizer plot loads: 0 (controls), 0.6, 3.0 and 15 kg N plot^−1^ (Table 1). A full factorial design would have resulted in very low and super-high local fertilizer concentration treatments, with the super-low treatment not yielding a sufficient difference in vegetation mass from the control and the super-high treatment causing the destruction of vegetation. Therefore, these two extreme treatments were not applied (Table 1).

**Table 1:** Fertilization treatments applied in the field fertilization experiment

Local nitrogen (N) concentration (g N m^−2^)	Plot fertilizer load (kg N plot^−1^)		
	2 × 2 m (*n* = 25)^a^	10 × 10 m (*n* = 5)^a^	50 × 50 m (*n* = 1)^a^
Control	0	0	0
1.2	–	0.6	3
6 g	0.6	3	15
30	3	15	–

The experiment involved the treatment of 50 × 50- m plots (*n* = 30) in such a way that the scale of fertilizer application and the local (within-patch) fertilizer concentration were independently varied for a given amount of fertilizer supplied to the plot as a whole. The treatments consisted of three different scales of fertilizer patchiness (columns), combined with four different within-patch fertilizer concentrations (rows) and three fertilizer loads per plot (diagonals). Two treatment combinations were not applied to avoid either very low or very high within-patch fertilizer concentrations

^a^Scale of patchiness: patch size with the number of patches per plot given in parenthesis

A commercial 3 N:2 P:1 K fertilizer was used, but for convenience fertilizer loads are expressed in terms of N supply per square meter in this paper. Fertilization, spread by hand, was initiated in December 2004, and the same within-plot areas (patches) were re-fertilized in December 2006. Plot corners and patches were permanently marked using iron pegs.

### Exclosure side experiment

The herbaceous off-take by large herbivores in relation to fertilizer concentration was determined by using the movable cage method (McNaughton et al. 1996; Prins and Beekman 1989) in a side experiment (within 500 m distance of the large experiment). Cages (1.0 × 1.5 × 0.6 m) were constructed from welded steel mesh and the tops covered with wire netting. Treatments consisted of the fertilization of 10 × 10-m plots in January 2006 using the same fertilizer stock and local fertilizer concentrations used in the large experiment. Treatments were replicated three times and randomly allocated to plots. The cages were first placed on treatment areas in February 2006, after which the aboveground biomass was regularly determined inside and outside of the grazer cages from six readings with a standard disc pasture meter (Waldram et al. 2008). The calibration by Zambatis et al. (2006) was used to convert disc settling height readings to biomass [kg dry matter (DM) ha^−1^]. During the growing season, measurements were taken and cages subsequently moved every 4–6 weeks depending on the growth rate of the herbaceous layer, while the time interval between readings was relaxed during the dry season when growth ceased. Off-take was calculated as the accumulated difference between the biomass inside and outside of the cages per plot.

### Tree measurements

Because of its local dominance, *C. mopane* was chosen as the focal tree species. In each plot 20 *C. mopane* trees of >1 m in height were selected and marked with aluminum tags. In the controls and whole-plot fertilizer treatments, *C. mopane* trees closest to 20 points evenly spaced over the plot area were selected. In heterogeneous treatments (i.e. the 2 × 2-m and 10 × 10-m patch scales), ten trees with stems within a 2-m distance of fertilized patches and ten trees evenly spaced stratified over the unfertilized plot area (>2 m distance from fertilized patches) were randomly selected. Four shoots on each of the marked trees were randomly selected by selecting the closest shoot tip to the top-end of a 1.35-m-long rod held against the canopy in the four compass directions around the canopy. Branches were marked with coded aluminum rings, which were positioned on shoots to include at least the previous 2004/2005 season’s (first fertilizer application season) growth increment. A thickened growth girdle on *C. mopane* shoots facilitated the identification of transitions between growing seasons. At the end of the experiment in 2008, all marked trees were visually assessed for the severity of large herbivore impact (mainly elephant; Pretorius et al. 2011) on canopies using the eight-point scale of Walker (1976).

The height of marked trees was calculated from digital photographs taken in 2006. At the end of the experiment in 2008, a selection of trees was re-measured to determine changes in tree height relative to canopy impact scores. In addition, the projected tree cover per plot was determined from digital aerial photographs taken from a microlight aircraft during the 2006/2007 growing season. The outlines of tree canopies in plots were mapped from the aerial photographs with the aid of GIS software (ArcView 3.3; Environmental Systems Research Institute, Redlands, CA). The tree cover of plots was expressed as the percentage of plot surface covered by tree canopies after geo-referencing the maps.

### Chemical analyses of leaves

Leaf samples from *C. mopane* trees and the two most prominent grass species, *U. mosambicensis* and *B. radicans*, were collected during the growing seasons of 2005/2006, 2006/2007 and 2007/2008. The N and P concentrations of the leaf samples were analyzed at the laboratory of the Resource Ecology Group, Wageningen University, The Netherlands. For *C. mopane*, five fully expanded leaves were randomly collected from the canopies of the marked trees. For *U. mosambicensis* and *B. radicans*, sub-samples were collected from mature plants nearest to the 20 points stratified over four transects dissecting the plot area. In homogeneous treatments (i.e. the control and 50 × 50-m fertilized plots), two pooled samples were analyzed per species per plot. In heterogeneous treatments, samples were pooled for leaves collected from the plants in and outside of the fertilized patches. Leaf samples were air dried in paper bags in a well-ventilated, shaded room before analysis. Prior to milling (1-mm sieve), *C. mopane* leaves were dried to a constant weight at 60 °C and then weighed. After destruction with a mixture of H_2_SO_4_, selenium and salicylic acid (Novozamsky et al. 1983), the N and P concentrations of the samples were measured in a San-plus autoanalyzer (Skalar Analytical B.V., Breda, the Netherlands)

### Herbaceous aboveground biomass

The aboveground biomass of the herbaceous layer in the 30 plots was assessed using a combination of the dry-weight-rank (DWR) and comparative-yield (CY) methods (Dekker et al. 2001; Friedel et al. 1988) In the DWR method, based on visual inspection, the herbaceous biomass contribution of the three most dominant species are ranked per quadrate. The ranks are assigned multipliers derived from empirical studies and then weighed according to the total biomass of individual quadrates (derived from the CY method). The CY method entails that the total herbaceous biomass per quadrate is assessed on a 10-point scale, which is then calibrated against actual cut-and-dry data.

The herbaceous standing crop was assessed towards the end of the growing season in 100 quadrates per plot, with each quadrate measuring 0.25 m^2^ (0.5 × 0.5 m) in size. These quadrates were stratified over nine evenly spaced transects dissecting the 50 × 50-m plot surface. In the 10 × 10-m and 2 × 2-m patch treatments, 25 quadrates were allocated to the fertilized patches, and the remaining 75 quadrates were stratified in the spaces between the fertilized patches, but allowing for a 2-m buffer around the fertilized patches. In the 2 × 2-m and 10 × 10-m patches, quadrates were assessed in the middle of the sub-plot, while an additional four estimates were recorded approximately 1 m inside the corners of the 10 × 10-m patches. For calibration purposes (CY method), the herbaceous aboveground biomass in seven quadrates per plot was clipped close to the ground (approx. 2 cm height) at the same time as the herbaceous assessment and dried to constant weight at 70 °C. Calibration quadrates were positioned in the buffer area surrounding the plots. The calibration dataset per sample year consisted of >200 data points, and separate calibration curves were calculated per observer per sample year. Calibration (power) functions had *R*^2^ values ranging from 0.90 to 0.96.

During the herbaceous assessment, the presence or absence of grazing signs and uprooted grass tuft remains, a sign of elephant grazing, were recorded.

### Plot-level estimates

In heterogeneous treatments, plot-level estimates of leaf N and P concentrations, net tree shoot growth and leaf mass, herbaceous biomass and proportion of trees impacted were calculated per plot from within-patch and outside-patch average values, corrected for the fraction of the total plot area covered by these areas. For controls and whole-plot treatments plot, averages were used.

### Statistical analysis

#### Local responses

Linear mixed models (LMMs) were used to test for the effects of fertilizer concentration and scale on the leaf N and P concentrations of *C. mopane* trees and *U. mosambicensis* and *B. radicans* grasses, tree shoot growth and herbaceous biomass, because both random factors (block) and repeated measures (sample year) can be included in the model. In heterogeneous treatments, responses in fertilized patches and outside patches were tested separately. Shoot growth measures in the marked *C. mopane* trees consisted of the net shoot length and net leaf mass per shoot and were a total of all shoot lengths and leaf mass per tree. Leaf N and P concentrations were arcsine transformed, biomass data were logarithmically transformed and *C. mopane* shoot data were square root transformed prior to analyses. For the tree data, trees were nested under plots, and plots were nested under experimental blocks. In all models, block was treated as a random factor. To statistically account for the suppressing effect of high tree cover on herbaceous biomass, the tree cover of plots was entered as a covariate in the herbaceous biomass model [the herbaceous aboveground biomass (kg DM ha^−1^) in control and non-fertilized areas of treated plots was negatively related to the percentage tree cover of plots (2005/2006 season: biomass = 1864.2 − 26.8 × tree cover; *F*_1,23_ = 12.3, *P* = 0.002; 2006/2007 season: biomass = 1326.3 − 17.3 × tree cover; *F*_1,23_ = 5.7, *P* = 0.025)].

Bonferroni multiple comparisons were used to differentiate between group means. Analysis of variance (ANOVA) was used to test for fertilization effects on annual herbaceous off-take and aboveground biomass production.

Binomial response data were analyzed using generalized linear models (GLMs). Within-patch fertilizer concentration and scale of patchiness were treated as factors in the models. Response variables were quadrates grazed/not grazed, quadrates with uprooted tufts present/not present and trees impacted/not impacted. The number of initially marked shoots lost by the end of the experiment per tree was also related to treatment factors in a GLM using a Poisson distribution. Separate tests were conducted for trees and quadrates within and outside of the fertilized patches. Block was entered as a random factor in GLMs.

Spearman correlations were performed to describe relationships between elephant impact scores, number of shoots lost per tree and shoot growth.

#### Plot-level responses

Mixed linear models were used to relate plot-level responses in leaf N and P concentrations, herbaceous biomass and net tree shoot growth to treatment factors. Treatment factors consisted of the fertilizer load that plots received and the grain size (scale) at which fertilizer was supplied. Block was entered as a random factor, with sample season as the repeated variable. The effect of treatment factors (fertilizer load and scale) on herbaceous aboveground net primary production for the 2006/2007 season was tested using ANOVA with block as a random factor.

All analyses were performed in SPSS version 15 (IBM Corp., Armonk, NY).

## Results

### Local leaf quality responses of trees and grasses to scale

**Fig. 1:**
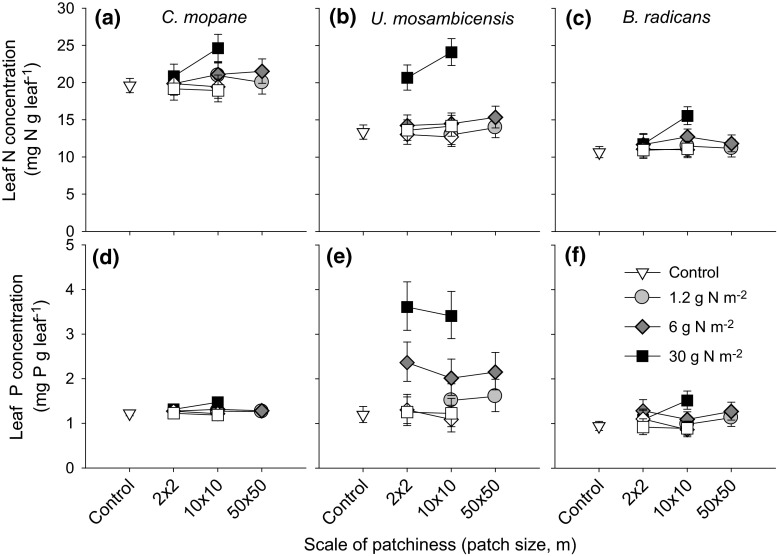
The responses of leaf nitrogen (*N*; **a**–**c**) and leaf phosphorus (*P*; (**d**–**f**) concentrations of the tree *Colophospermum mopane* and grasses *Urochloa mosambicensis* and *Bothriochloa radicans*, to the scale of nutrient patchiness and the within-patch fertilizer concentration. The results are given for plants within the fertilized patches (*filled symbols*) and outside of these patches (>2 m distance from patch edges,* open symbols*). Means and 95 % confidence intervals (CIs) are given

Both grass species and the tree species responded to fertilization by accumulating higher N and P concentrations in their leaves (Fig. 1; Electronic Supplementary material (ESM) Table S1, main concentration effect). Supporting the first hypothesis, the leaf N and P concentrations of *C. mopane* trees were also significantly different between plots of different patch sizes, with stronger responses of leaf N concentration in larger patches (10 × 10 m) than in smaller patches (2 × 2 m) (Fig. 1; ESM Table S1, concentration × patch size interaction). However, in contrast to expectations, the responses of the grasses also tended to be stronger with increasing patch size. For the highest fertilizer concentration, leaf N concentrations of *U. mosambicensis* and *B. radicans* were higher in 10 × 10-m patches than in 2 × 2-m patches (Fig. 1; ESM Table S1, concentration × patch size interaction). The responses of leaf N and P concentrations were consistent over the 3 years of study (ESM Figs. S1, S2).

The responses were confined to the fertilized patches only: leaf N and P concentrations of both trees and grasses outside of the fertilized patches (i.e. >2 m distance from fertilized patches) were not affected by fertilization in nearby patches (LMMs; *P* > 0.05; Fig. 1).

### Local herbivore impact in relation to scale

**Fig. 2:**
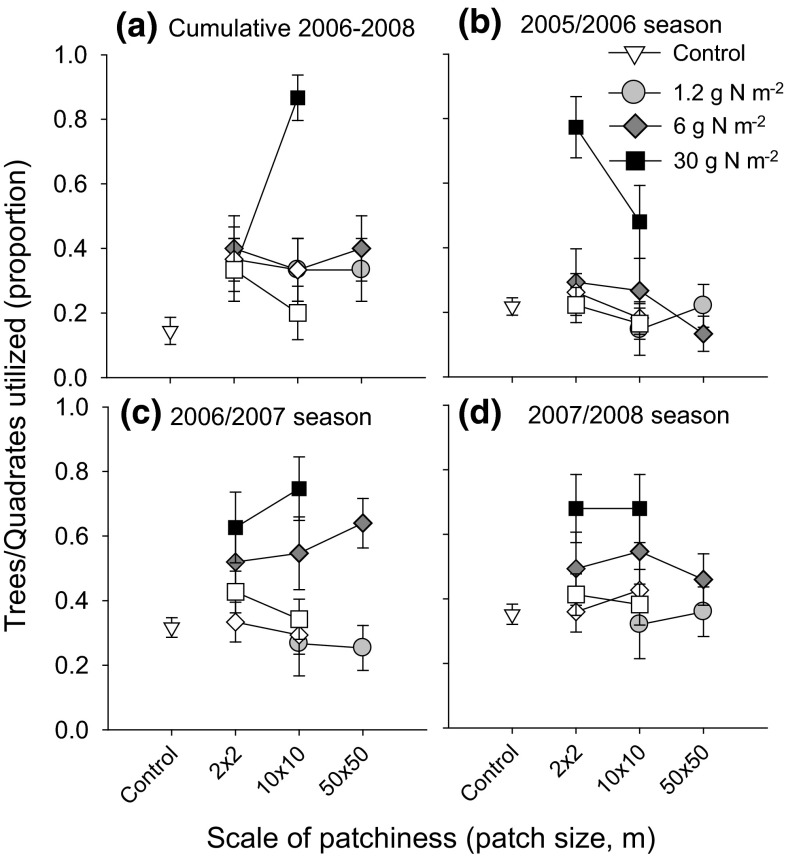
Estimates of the proportion of *C. mopane* trees impacted by elephants from 2006 to 2008 (**a**) and the proportion of quadrates showing signs of grazing by large herbivores for the 2005/2006, 2006/2007 and 2007/2008 seasons (**b**,** c**, **d**, respectively). The results are given for plants within the fertilized patches (*filled symbols*) and outside of these patches (>2 m distance from patch edges, *open symbols*). Means and 95 % CIs are given

In fertilized patches, the impact of browsers on the tree layer differed between the scale–fertilizer concentration treatments (Wald Chi-square = 105.064, *n* = 300, *P* < 0.001). In total, 87 % of the marked *C. mopane* trees in the 10 × 10-m patches, fertilized at a rate of 30 g N m^−2^, showed browser impact, which was double the impact in the 2 × 2-m patch treatment fertilized at the same high concentration (Fig. 2a). In the control treatment, only 18 % of the trees showed signs of browser damage. The proportion of impacted trees in other treatments tended to be higher but was not significantly different from that of controls (Bonferroni adjusted *P* > 0.05; Fig. 2a). The proportions of trees impacted by browsers outside the fertilized patches were also not different from those of the control (*P* > 0.05).

Of the trees in the 10 × 10 m—30 g N m^−2^ patches, 20 % were estimated to have >25 % of the canopy volume removed by browsers, compared to only 1 % of trees in control plots, indicating that not only the frequency of trees impacted increased, but also the intensity of utilization per tree. The impact severity scores showed a negative correlation (Spearman, *r* = −0.49, *n* = 44, *P* = 0.001) with tree height change over the study period (2006–2008). Impacted trees decreased in height, while the height of trees not impacted or only lightly impacted stayed approximately constant over the study period. The number of marked shoots lost per marked tree was positively correlated with impact severity (Spearman, *r* = 0.17, *n* = 600, *P* < 0.001).

The estimated grazing intensity intensified with increasing fertilizer concentration in patches (2005/2006 season: Wald Chi-square = 102.0, *n* = 1125, *P* < 0.001; 2006/2007 season: Wald Chi-square = 139.5, *n* = 1125, *P* < 0.001; 2007/2008 season: Wald Chi-square = 76.8, *n* = 1125, *P* < 0.001). The number of quadrates that showed signs of grazing increased from about 30 % in the controls to about 70 % in patches receiving 30 g N m^−2^. The interaction between grazing intensity and patch size was variable between years. The 2 × 2-m fertilized patches were heavily grazed in the first year of study, but less so in subsequent years (Fig. 2b). In the second relatively wet year, grazing intensity tended to increase with patch size for the two higher levels of fertilization, but this trend leveled off in the last dry year (Fig. 2b).

Grazers utilized the fertilized patches very selectively: grazing intensities in the non-fertilized areas situated in between the fertilized 2 × 2- and 10 × 10-m patches did not differ from grazing frequencies in the control plots.

**Fig. 3:**
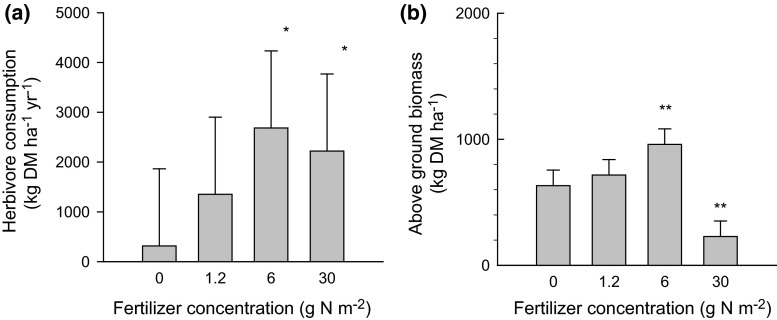
Results of a movable exclosure experiment showing the estimated annual herbaceous off-take by large herbivores (**a**) and the aboveground biomass measured outside the exclosure cages at the end of the growing season (April 2007) (**b**) in relation to 10 × 10-m plots fertilized at different fertilizer concentrations. *DM* Dry matter. Means and 95 % CIs are given. *Asterisks above bars* indicate least square differences from control values (0 g N m^−2^), *P < 0.05, **P < 0.01

In the 2006–2007 season, the amount of herbaceous off-take by large herbivores was also affected by the local fertilizer concentration (exclosure experiment; ANOVA, *F*_3,8_ = 4.3, *P* = 0.044). Relative to controls, the off-take by herbivores increased by threefold in the 1.2 g N m^−2^ treatment, by sevenfold in the 6 g N m^−2^ treatment and by sixfold in the 30 g N m^−2^ treatment (Fig. 3a). The aboveground biomass in March 2007 (peak biomass) outside of exclosure cages differed between fertilizer treatments (ANOVA, *F*_3,8_ = 13, *P* = 0.002) and was on average 64 % lower in the 30 g N m^−2^ treatment than in the 0 g N m^−2^ treatment (Fig. 3b). No differences in total herbaceous off-take by large herbivores (ANOVA, *P* > 0.05) were detected between fertilizer treatments in the April 2007 to March 2008 period, when poor rainfall was received.

### Local net biomass responses of the tree and herbaceous layers

In trees, marked shoots were differentially lost across treatments. By the end of the experiment in 2008, trees in the 10 × 10-m patches fertilized at 30 g N m^−2^ lost on average 1.8 of four marked shoots per tree, compared to only 0.9 shoots lost on average per control tree (Bonferroni adjusted *P* < 0.001). Shoot loss in other treatments were not different from shoot loss in the controls (*P* > 0.05).

**Fig. 4:**
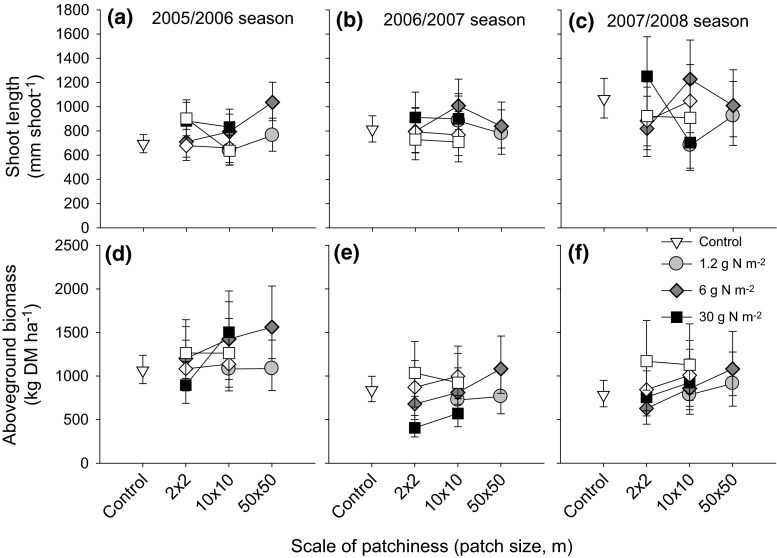
Net shoot length per marked *C. mopane* tree (**a**–**c**) and aboveground herbaceous biomass (**d**–**f**) in response to scale of nutrient patchiness and within-patch fertilizer concentration for the 2005/2006, 2006/2007 and 2007/2008 seasons, respectively. The responses for plants within fertilized patches (*filled symbols*) and outside of these patches (not fertilized, *open symbols*) are shown. Means and 95 % CIs are given

The net biomass responses of trees and grasses are the net effect of increased growth due to fertilization and off-take by large herbivores. Taking shoot losses into account, both net shoot length and shoot mass increased at higher local fertilizer concentration (Fig. 4a; ESM Table S2). Both parameters showed changes over the years that interacted with patch size. The year × fertilizer concentration × patch size interaction (ESM Table S2) for shoot length was strongly influenced by shoot growth of trees in the 10 × 10-m patch–30 g N m^−2^ treatment which declined from relatively high values in the 2005/2006 season to below-control values in the 2007/2008 season (Fig. 4a). The net leaf mass of trees followed a similar pattern (ESM Table S2).

In the heterogeneous treatments, trees outside of the fertilized patches showed an interaction between year and the scale of patchiness (*F*_4,320_ = 2.4, *P* = 0.050; Fig. 4a). This interaction was influenced by higher tree growth outside of the fertilized patches in the 2 × 2 m scale of patchiness—30 g N m^−2^ treatment in the 2005/2006 season, which levelled off in ensuing seasons (Fig. 4a).

For the herbaceous aboveground biomass, the overall effect of fertilizer concentration was not significant, indicating that over all years and treatments the higher biomass production due higher nutrient availability was completely offset by herbivore consumption (ESM Table S2). Over the years, herbaceous biomass tended to be higher in the larger fertilized patches and lower in the smaller fertilized patches (Fig. 4b), leading to a significant fertilizer x patch size interaction (ESM Table S2). Remarkably, in the heterogeneous treatments, the herbaceous biomass in between the fertilized 2 × 2 m and 10 × 10 m patches tended to be higher than in the fertilized patches themselves (Fig. 4b). Biomass in these unfertilized areas was unrelated (linear mixed models, *P* > 0.05) to fertilizer concentrations or scale of patchiness (Fig. 4b).

### Plot-level responses to scale

The plot-level responses of *C. mopane* leaf N and P concentrations, which were corrected for the relative area covered by fertilized patches and unfertilized interspaces in plots, were either slightly higher or unrelated to the fertilizer load (ESM Table S3). Concentrations were unrelated to the grain size at which fertilizer was supplied to plots (LMM, *P* > 0.05; ESM Table S3). The leaf N concentration of the grasses *U. mosambicensis* (mixed linear model, *F*_3,18_ = 9.789, *P* < 0.001) and *B. radicans* (*F*_3,13_ = 3.521, *P* < 0.05) responded to fertilizer load and were higher in the 15 kg N plot^−1^ treatment (Bonferroni adjusted *P* < 0.01). Neither species responded to scale (*P* > 0.05; ESM Table S3), although in the relatively wet season 2006–2007, leaf N concentrations for the highest fertilization level tended to be higher at the largest patch size (50 × 50-m patch; ESM Fig. S3).

Plot-level *U. mosambicensis* leaf P concentration, however, did increase with fertilizer load (*P* < 0.001) and was affected by scale (*P* < 0.01), but the load × scale interaction was not significant (*P* > 0.05; ESM Table S3). For the same fertilizer load, the P concentration of *U. mosambicensis* leaves was higher (Bonferroni adjusted, *P* < 0.05) in the 50 × 50-m grain size plots than in either the 10 × 10-m or 2 × 2-m grain size plots, which did not differ from each other (Bonferroni adjusted, *P* > 0.5; ESM Fig. S4). Overall, plot-level *B. radicans* leaf P concentration was unresponsive to treatments (mixed linear model, *P* > 0.05) but varied over the years, resulting in lower values with increasing patch size, an effect in the opposite direction compared to *U. mosambicensis*.

**Fig. 5:**
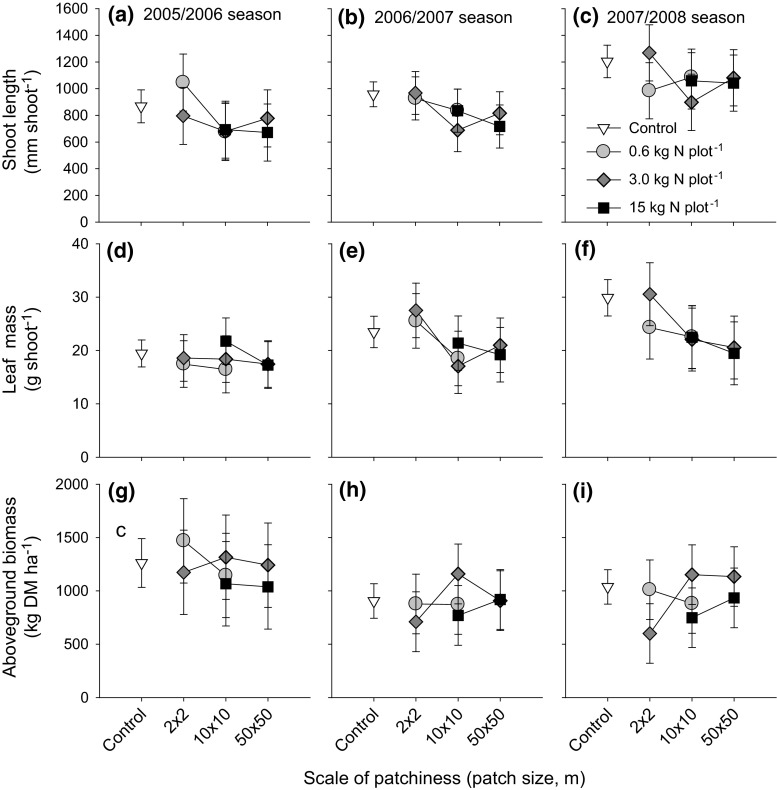
Plot level estimates of shoot length (**a**–**c**) and leaf mass of the tree, *C. mopane* (**d**–**f**) and aboveground herbaceous biomass (**g**–**i**) to different fertilizer loads and patch sizes at which fertilizer was supplied to the 50 × 50-m plots for the 2005/2006, 2006/2007 and 2007/2008 seasons, respectively. Means and 95 % CIs are given

Plot-level estimates of the tree net shoot length, leaf mass per shoot and aboveground herbaceous biomass were not significantly related to fertilizer load or the scale of patchiness (mixed linear models, *P* > 0.1; ESM Table S4). However, opposite trends for trees and grasses were discernible (Fig. 5). Over the years, average tree leaf mass over the entire plot decreased with increasing patch size. At larger fertilization scales, leaf mass at plot scale tended to be smaller in fertilized plots than in unfertilized control plots (Fig. 5b). Although not significantly different, plot-level averages of the proportion of trees (area corrected) impacted by browsers over the study period was almost twofold higher in fertilized plots than in the unfertilized control plots (Fig. S5). For the herbaceous layer, plot level biomass estimates tended to increase with plot size (Fig. 5c). At the highest fertilizer load per plot, herbaceous biomass tended to be lower rather than higher than that in the unfertilized plots.

## Discussion

Previous studies have demonstrated scale effects in plant communities (Hutchings et al. 2003; Wijesinghe et al. 2005) or scale effects of grazing resources for a grazer assemblage (Cromsigt and Olff 2006; Cromsigt et al. 2009; Ritchie and Olff 1999), but to our knowledge our study is the first to investigate how the scale of a patchily distributed soil resource influences interactions between trophic levels. Due to scale-dependent foraging of plants and herbivores, we expected that fertilizing at different patch sizes would result in scale-dependent shifts in the balance between grasses and trees in this savanna ecosystem. However, if such scale-dependent shifts occurred, they were minor and not according to expectation. The main reason for this result is that localized plant responses to elevated nutrition were very effectively utilized by the community of large herbivores, irrespective of the scale of fertilization. The combined effect was that the effects of fertilization were completely leveled off by grazing and browsing, leading to even lower biomass than in control areas that remained unfertilized. Herbaceous biomass data suggest that grasses profited slightly from fertilization at larger patch sizes, which is in contrast to expectations. In the following sections we discuss the scale-dependent plant and herbivore responses and their interactions that underlie these overall effects.

### Local responses of trees and grasses to fertilization and to scale

Increases in local nutrient concentrations resulted in increases in leaf N and P concentrations of both trees and grasses inside the fertilized patches. Based on scaling theory, we expected grasses to profit particularly from the smaller patches and trees particularly from the larger patches—but this was not the case. At the highest local fertilizer concentration, where responses were the strongest, both trees and grasses had higher leaf N concentrations at larger (10 × 10 m) rather than smaller (2 × 2 m) patch sizes. These results suggest that trees and grasses were less different than hypothesized. Both life forms are well able to forage for localized resources (Einsmann et al. 1999), and grasses also have a root system that extends well beyond their own canopy (Pecháčková et al. 2003). Consequently, grasses rooting in the smaller 2 × 2-m patches probably also had roots outside of the fertilized patches and acquired less nutrients than the grasses in the 10 × 10-m patches for which this applied only to plants at the edges of the patch. However, edge effects were narrow. Trees outside the patches were generally unresponsive to fertilization, probably reflecting the short stature of trees (3.1 ± 1.6 m; mean ± standard deviation) in the study area. In the case of grasses, fertilization-induced changes in leaf greenness and aboveground biomass rapidly dissipated beyond patch boundaries (effects disappeared within 0.2 m from patch edge; C. van der Waal, personal observation), suggesting that the effective horizontal root range of grasses was small.

### Local responses of browsers and grazers to fertilization and to scale

Grazers and browsers were very effective in finding forage of higher quality (i.e. containing higher concentrations of nutrients). Strikingly, the patches that peaked in leaf N concentrations (Fig. 1) also peaked in herbivore utilization (Fig. 2). This well-known response of herbivores (e.g. Pretorius et al. 2011) was of far greater importance than the hypothesized scale-dependent foraging of browsers and grazers. Irrespective of the size of the fertilized patch, the impact of browsing on trees was slightly higher than control levels and considerably higher in the 10 × 10-m patch at highest local fertilization concentration, which corresponds with trees with the highest leaf nutrient concentration. For the herbaceous layer, on average 80 % of the smallest 2 × 2-m patches at the highest fertilizer level were immediately located by the herbivores in the first year, and a considerable number were grazed and uprooted, most likely by the largest herbivore, namely, the elephant. In the second relatively wet season, grazer impact intensified at larger patch scales. Regarding the overall impact of the herbivore community, we conclude that grazers did not focus predominantly at smaller scales, thereby refuting hypothesis 2. Browsers avoided the smaller scales, but they did seem to respond foremost to leaf nutrient concentration of the trees. We cannot rule out that individual herbivores may have responded to the scale of forage resources (Cromsigt et al. 2009; Kohi et al. 2011; Laca et al. 2010; Pretorius et al. 2011), but overall scale effects on the plant community were mixed at best.

Results from the exclosure experiment and data on shoot loss of browsed trees indicate that off-take by herbivores was considerable. At the highest fertilization level, the marginal increase in plant growth in this semi-dry system may have been small relative to the strong herbivore response to elevated leaf nutrient concentrations. Indeed, the net effect of fertilization and herbivory became increasingly negative over the years of study, particularly in the most heavily impacted patches. Consequently, on average, tree shoot growth and herbaceous biomass in fertilized patches was even lower than that in adjacent unfertilized areas. In an herbivore-dominated system such as the African savanna (Asner et al. 2009; Young et al. 2013), our general notion of how patchy fertilization profits plant growth must be reconsidered.

Although we do not have scale-dependent measures, grazer off-take per square meter may have been larger with smaller patches. For example, 2 × 2-m patches were often found to be completely overgrazed but this was not the case for entire 10 × 10-m patches (C. van der Waal, personal observation), which may explain why end-of-season herbaceous biomass in smaller fertilized patches was lower than that in control areas which remained unfertilized (Fig. 4b). It could also explain the trend that herbaceous biomass in fertilized patches increased slightly with patch size. At the same time, grasses and trees are known to compete strongly for belowground resources (especially water) (Knoop and Walker 1985; Ludwig et al. 2004; Riginos 2009; Stuart-Hill and Tainton 1989; van der Waal et al. 2009). The enhanced tree growth in the fine-scale treatment may have been caused by a release from grass competition facilitated by selective overgrazing of the small-scale patches.

### Consequences of scale at the community level

How did the localized responses of trees, grasses, browsers and grazers add up to community responses at plot scale? We hypothesized that, for a similar overall nutrient load per plot, finer scaled fertilization would profit the herbaceous layer, and larger scaled fertilization the tree layer. However, due to the very effective utilization of the fertilized patches by the herbivores, the profits to the plant community were completely eliminated. In fact, overall biomass measures tended to be smaller rather than larger in fertilized plots compared to unfertilized controls. The 10 × 10-m patch treatment may represent local nutrient hotspots that are often observed in savannas—for example, created by termitaria, animal burrows, abandoned kraals or bomas or beneath trees (Belsky 1994; Pretorius et al. 2011; van der Waal et al. 2011b). These nutrient hotspots may supply scarce nutrients in concentrated form that is not elsewhere available to herbivores (Grant and Scholes 2006; Pretorius et al. 2011). Our results clearly suggest that the herbivore community will profit from such patches rather than the primary producers.

Scale-dependent effects were minor, and trends were opposite to those hypothesized. After 3 years, the tree community tended to perform worse, and the herbaceous plant community better when the same amount of fertilizer was supplied coarse-scaled rather than fine-scaled (Fig. 5). How can this counterintuitive result be understood? The same amount of fertilizer concentrated in a small number of patches affects only a small proportion of the herbaceous community, which is subsequently overgrazed, as discussed above. Spreading the same amount of fertilizer over a larger patch area leads to less growth stimulus but also to less herbivory impact (Milchunas and Lauenroth 1993). The leaf mass response of the trees to scale in the last couple of years of the study was exactly opposite to the herbaceous biomass response to scale (compare Fig. 5b, c), suggesting that the tree community performed relatively well from fertilization at smaller scales due to reduced competition from the grasses. Another possibility is that trees profited directly from the fertilizer itself if, at the highest concentrations, not all nutrients were taken up by the grasses and leaked to deeper soil layers with a higher presence of roots of trees.

Based on the 3 years of our study, it is too early to tell whether fertilization in patches of different size leads to shifts in the tree–grass balance in this savanna ecosystem. Effects are small and still transient and are partly affected by season-specific rainfall. However, the trend which is discernible is opposite to the trend hypothesized based on scaling theory predicting the scales at which plants and animals preferentially forage for resources. We conclude that the scale of resource patchiness may ultimately affect the local partitioning of resources between co-existing plant growth forms, such as trees and grasses in the savanna, but that these responses may be counterintuitive and can only be understood if bi-trophic interactions are taken into account.

## Electronic supplementary material

Below is the link to the electronic supplementary material.

Supplementary material 1 (DOCX 267 kb)
