# Supplementary material for: Scale-dependent bi-trophic interactions in a semi-arid savanna: how herbivores eliminate benefits of nutrient patchiness to plants
Source: Oecologia. 2016 Apr 19;181:1173–85. doi: 10.1007/s00442-016-3627-0 (PMC4954840; doi:10.1007/s00442-016-3627-0)
Supplement: Supplementary file 1 — Supplementary material 1 (DOCX 267 kb) [file 442_2016_3627_MOESM1_ESM.docx]

**Electronic Supplementary Material**

**Herbivores forage for plants, which forage for nutrients: Scale-dependent bi-trophic interactions in a semi-arid savanna**

*Cornelis van der Waal, Hans de Kroon, Frank van Langevelde, Willem F. de Boer, Ignas M.A. Heitkönig, Rob Slotow, Yolanda Pretorius, Herbert H.T. Prins*

**Table S1.** Linear mixed model tests for leaf N, and leaf P concentrations of *C. mopane* trees, and *U. mosambicensis* and *B. radicans* grasses in fertilized patches (dependent variables) as affected by within-patch fertilizer concentration and fertilizer patch size (fixed factors). Sampling was repeated in 2006, 2007 and 2008 (within-subjects factor)

| Source of variation | ***C. mopane*** | |  | ***U. mosambicensis*** | | | |  | | | ***B. radicans*** | | |
| --- | --- | --- | --- | --- | --- | --- | --- | --- | --- | --- | --- | --- | --- |
|  | **d.f.** | **F** |  | **d.f.** | **F** |  | | | **d.f.** | | | | **F** |
| Leaf nitrogen | | | | | |  | | |  | | | |  |
| *Within subjects* |  |  |  |  |  |  | | |  | | | |  |
| Year | 2,43 | 51.8 |  | 2,44 | 163.6*** |  | | | 2,25 | | | | 22.9*** |
| Year * Concentration | 6,43 | 0.9 |  | 6,44 | 8.1*** |  | | | 6,24 | | | | 1.0 |
| Year * Patch size | 4,43 | 1.5 |  | 4,44 | 0.8 |  | | | 4,25 | | | | 0.6 |
| Year * Conc. * Patch size | 8,43 | 0.5 |  | 8,44 | 1.0 |  | | | 7,24 | | | | 1.2 |
|  |  |  |  |  |  |  | | |  | | | |  |
| *Between subjects* |  |  |  |  |  |  | | |  | | | |  |
| Concentration | 3,26 | 21.9*** |  | 3,25 | 66.2*** |  | | | 3,15 | | | | 12.1*** |
| Patch size | 2,25 | 18.1*** |  | 2,25 | 2.2 |  | | | 2,16 | | | | 6.0* |
| Conc. * Patch size | 4,25 | 3.2* |  | 4,25 | 2.9* |  | | | 4,15 | | | | 1.1 |
|  |  |  |  |  |  |  | | |  | | | |  |
| Leaf phosphorous | | | | | | |  | | |  | |  | |
| *Within subjects* |  |  |  |  |  |  | | |  | | | |  |
| Year | 2,45 | 12.4*** |  | 2,41 | 18.5*** |  | | | 2,32 | | | | 16.8*** |
| Year * Concentration | 6,45 | 0.2 |  | 6,41 | 5.1** |  | | | 6,30 | | | | 2.3 |
| Year * Patch size | 4,44 | 0.4 |  | 4,41 | 3.0* |  | | | 4,30 | | | | 1.3 |
| Year * Conc. * Patch size | 8,45 | 0.3 |  | 8,41 | 2.2* |  | | | 7,30 | | | | 0.8 |
|  |  |  |  |  |  |  | | |  | | | |  |
| *Between subjects* |  |  |  |  |  |  | | |  | | | |  |
| Concentration | 3,28 | 9.7*** |  | 3,24 | 49.1*** |  | | | 3,23 | | | | 12.2*** |
| Patch size | 2,27 | 3.6* |  | 2,24 | 0.6 |  | | | 2,25 | | | | 2.1 |
| Conc. * Patch size | 4,27 | 1.0 |  | 4,24 | 0.1 |  | | | 4,22 | | | | 6.1** |
|  |  |  |  |  |  |  | | |  | | | |  |

*P<0.05; **P<0.01; ***P<0.001

**Table S2.** Linear mixed model tests for mean annual shoot length increase and leaf mass per shoot of *C. mopane* and herbaceous aboveground biomass in fertilized patches (dependent variables) as affected by within-patch fertilizer concentration and fertilizer patch size (fixed factors). Sampling was repeated in 2006, 2007 and 2008 (within-subjects factor). Tree cover (% of plot surface) was entered as a covariate in the herbaceous biomass model

| **Source of variation** | ***C. mopane* annual shoot length** | | |  | | ***C. mopane* annual leaf mass per shoot** | | |  | **Herbaceous aboveground biomass** | |
| --- | --- | --- | --- | --- | --- | --- | --- | --- | --- | --- | --- |
|  | **d.f.** | **F** |  | | **d.f.** | | **F** |  | | **d.f.** | **F** |
| *Within subjects* |  |  |  | |  | |  |  | |  |  |
| Year | 2,388 | 109.4*** |  | | 2,391 | | 96.6*** |  | | 2,39 | 39.3*** |
| Year * Concentration | 6,388 | 0.8 |  | | 6,392 | | 1.0 |  | | 6,39 | 4.4** |
| Year * Patch size | 4,387 | 8.3*** |  | | 4,390 | | 1.3 |  | | 4,39 | 0.4 |
| Year * Conc. * Patch size | 8,387 | 2.2* |  | | 8,390 | | 2.2* |  | | 8,39 | 0.3 |
|  |  |  |  | |  | |  |  | |  |  |
| *Between subjects* |  |  |  | |  | |  |  | |  |  |
| Concentration | 3,297 | 4.2** |  | | 3,298 | | 3.9* |  | | 3,21 | 1.3 |
| Patch size | 2,297 | 1.2 |  | | 2,297 | | 0.4 |  | | 2,21 | 1.9 |
| Conc. * Patch size | 4,297 | 0.7 |  | | 4,297 | | 0.4 |  | | 4,21 | 3.2* |
| Tree cover (covariate) | - | - |  | | - | | - |  | | 1,21 | 18.6*** |

*P<0.05; **P<0.01; ***P<0.001

**Table S3.** Linear mixed model test statistics for plot-average leaf N and leaf P concentrations of *C. mopane* trees, and *U. mosambicensis* and *B. radicans* grasses (dependent variables) as affected by fertilizer load per plot and the grain size (scale) at which fertilizer was supplied to plots (fixed factors). Sampling was repeated in 2006, 2007 and 2008 (within-subjects factor)

| **Source of variation** | ***C. mopane*** | |  | ***U. mosambicensis*** | | | |  | | | ***B. radicans*** | | | |
| --- | --- | --- | --- | --- | --- | --- | --- | --- | --- | --- | --- | --- | --- | --- |
|  | **d.f.** | **F** |  | **d.f.** | **F** |  | | | **d.f.** | | | | **F** | |
| Leaf N | | | | | |  | | |  | | | |  | |
| *Within subjects* |  |  |  |  |  |  | | |  | | | |  | |
| Year | 2,19 | 80.7*** |  | 2,20 | 128.0*** |  | | | 2,15 | | | | 25.2*** | |
| Year * Load | 6,19 | 0.1 |  | 6,20 | 1.0 |  | | | 6,17 | | | | 0.5 | |
| Year * Grain size | 4,19 | 1.1 |  | 4,20 | 0.9 |  | | | 4,16 | | | | 0.3 | |
| Year * Load * Grain size | 8,20 | 0.7 |  | 8,20 | 1.3 |  | | | 7,18 | | | | 3.8* | |
|  |  |  |  |  |  |  | | |  | | | |  | |
| *Between subjects* |  |  |  |  |  |  | | |  | | | |  | |
| Load | 3,17 | 3.4* |  | 3,20 | 8.4** |  | | | 3,15 | | | | 4.0* | |
| Grain size | 2,17 | 1.1 |  | 2,20 | 0.8 |  | | | 2,16 | | | | 1.1 | |
| Load * Grain size | 4,17 | 0.6 |  | 4,20 | 0.9 |  | | | 4,16 | | | | 1.3 | |
|  |  |  |  |  |  |  | | |  | | | |  | |
| Leaf P | | | | | | |  | | |  | |  | | |
| *Within subjects* |  |  |  |  |  |  | | |  | | | | |  |
| Year | 2,20 | 10.1** |  | 2,20 | 90.6*** |  | | | 2,14 | | | | | 34.3*** |
| Year * Load | 6,20 | 0.1 |  | 6,20 | 1.4 |  | | | 6,15 | | | | | 3.1* |
| Year * Grain size | 4,20 | 0.3 |  | 4,20 | 1.2 |  | | | 4,15 | | | | | 5.5** |
| Year * Load * Grain size | 8,21 | 0.5 |  | 8,20 | 1.3 |  | | | 7,12 | | | | | 10.1*** |
|  |  |  |  |  |  |  | | |  | | | | |  |
| *Between subjects* |  |  |  |  |  |  | | |  | | | | |  |
| Load | 3,21 | 1.7 |  | 3,20 | 14.7*** |  | | | 3,16 | | | | | 1.9 |
| Grain size | 2,20 | 0.5 |  | 2,20 | 5.4* |  | | | 2,16 | | | | | 2.3 |
| Load * Grain size | 4,20 | 0.5 |  | 4,20 | 1.2 |  | | | 4,17 | | | | | 1.0 |
|  |  |  |  |  |  |  | | |  | | | | |  |

*P<0.05; **P<0.01; ***P<0.001

**Table S4** Linear mixed model statistics for plot-averages of net shoot length and net leaf mass per shoot of *C. mopane* trees and herbaceous aboveground biomass (dependent variables) as affected by fertilizer load per plot and the grain size (scale) at which fertilizer was supplied to plots (fixed factors). Sampling was repeated in 2006, 2007 and 2008 (within-subjects factor). Tree cover (% of plot surface) was entered as a covariate in the herbaceous biomass model.

| **Source of variation** | ***C. mopane* net shoot length** | | |  | | ***C. mopane* net leaf mass per shoot** | | |  | **Herbaceous aboveground biomass** | |
| --- | --- | --- | --- | --- | --- | --- | --- | --- | --- | --- | --- |
|  | **d.f.** | **F** |  | | **d.f.** | | **F** |  | | **d.f.** | **F** |
| Year | 2,20 | 25.4*** |  | | 2,20 | | 16.1*** |  | | 2,20 | 32.9*** |
| Year * Load | 6,20 | 0.7 |  | | 6,20 | | 0.3 |  | | 6,20 | 1.3 |
| Year * Grain size | 4,20 | 0.9 |  | | 4,20 | | 1.3 |  | | 4,20 | 0.2 |
| Year * Load * Grain size | 8,20 | 0.4 |  | | 8,20 | | 0.7 |  | | 8,20 | 1.4 |
|  |  |  |  | |  | |  |  | |  |  |
| *Between subjects* |  |  |  | |  | |  |  | |  |  |
| Load | 3,18 | 2.3 |  | | 3,16 | | 1.3 |  | | 3,18 | 2.6 |
| Grain size | 2,18 | 0.9 |  | | 2,16 | | 0.5 |  | | 2,18 | 1.4 |
| Load * Grain size | 4,18 | 1.8 |  | | 4,16 | | 1.0 |  | | 4,17 | 0.3 |
| Tree cover | - | - |  | | - | | - |  | | 1,19 | 25.4*** |

*P<0.05; **P<0.01; ***P<0.001

Fig. S1.

**Fig. S1**. The leaf N concentration responses of (a) *U. mosambicensis,* (b) *B. radicans* and (c) *C. mopane* to the scale of nutrient patchiness and the within-patch fertilizer concentration for the 2005/2006, 2006/2007 and 2007/2008 seasons. The results are given for plants within the fertilized patches (filled symbols) and outside these patches (>2 m distance from patch edges, open symbols). Means and 95% confidence intervals

Fig. S2

**Fig. S2**. The leaf P concentration responses of (a) *U. mosambicensis,* (b) *B. radicans* and (c) *C. mopane* to the scale of nutrient patchiness and the within-patch fertilizer concentration for the 2005/2006, 2006/2007 and 2007/2008 seasons. The results are given for plants within the fertilized patches (filled symbol) and outside these patches (>2 m distance from patch edges, open symbols). Means and 95% confidence intervals are given

Fig S3.

**Fig. S3**. Plot-level leaf N responses of (a) *U. mosambicensis,* (b) *B. radicans* and (c) *C. mopane* to different fertilizer plot loads and patch sizes at which fertilizer was supplied to the 50 x 50 m plots for the 2005/2006, 2006/2007 and 2007/2008 seasons. Means and 95% confidence intervals are given

Fig. S4

**Fig. S4**. Plot-level leaf P responses of (a) *U. mosambicensis,* (b) *B. radicans* and (c) *C. mopane* to different fertilizer plot loads and patch sizes at which fertilizer was supplied to the 50 x 50 m plots for the 2005/2006, 2006/2007 and 2007/2008 seasons. Means and 95% confidence intervals are given
